# Supplementary material for: Splice-Junction-Based Mapping of Alternative Isoforms in the Human Proteome
Source: Cell Rep. Author manuscript; Available in PMC 2020 Jan 15. (PMC6961840; doi:10.1016/j.celrep.2019.11.026)
Supplement: 2 [file NIHMS1546469-supplement-2.zip › DF1/PXD009737/TestisGluC/TestisGluC_27_MAP3K11_AGEGPAWTKPHGTWIQMTHPP.pdf]

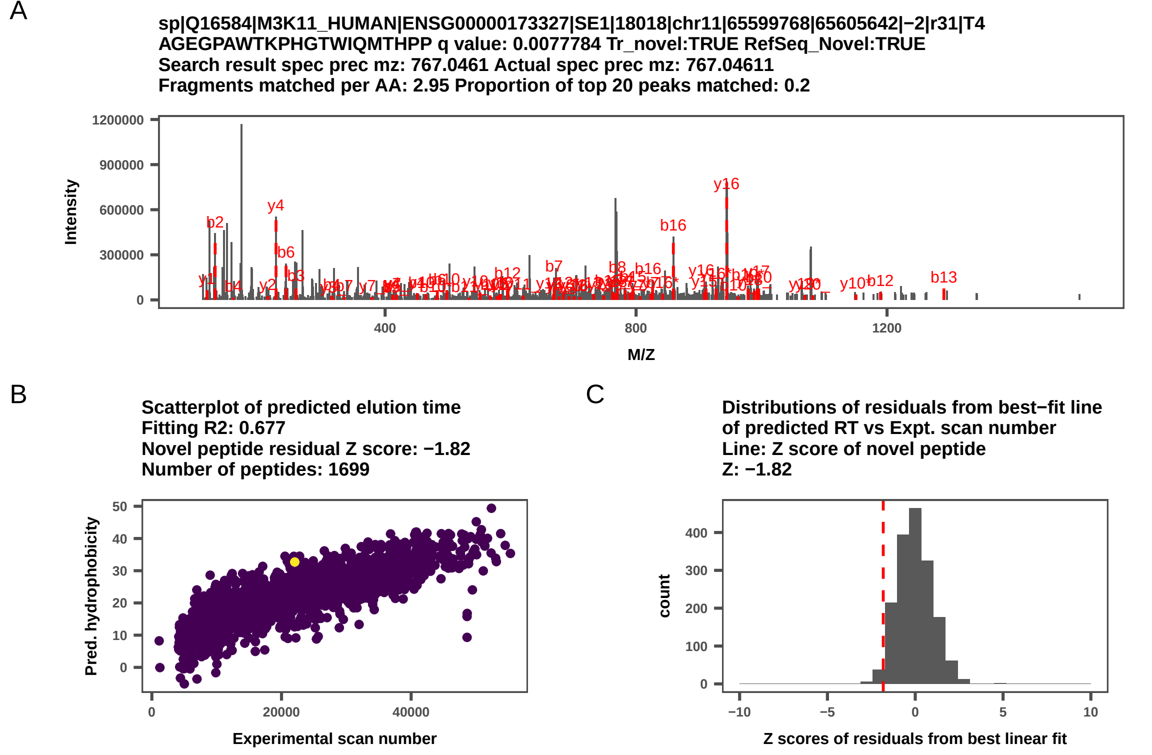
A
sp|Q16584|M3K11_HUMAN|ENSG00000173327|SE1|18018|chr11|65599768|65605642|−2|r31|T4
AGEGPAWTKPHGTWIQMTHPP q value: 0.0077784 Tr_novel:TRUE RefSeq_Novel:TRUE
Search result spec prec mz: 767.0461 Actual spec prec mz: 767.04611
Fragments matched per AA: 2.95 Proportion of top 20 peaks matched: 0.2
Intensity
1200000
900000
600000
300000
0
y16
y4
b2
b16
b6
b3
b7
b8
b12
b13
400
800
1200
M/Z
B
Scatterplot of predicted elution time
Fitting R2: 0.677
Novel peptide residual Z score: −1.82
Number of peptides: 1699
Pred. hydrophobicity
50
40
30
20
10
0
0
20000
40000
Experimental scan number
C
Distributions of residuals from best−fit line
of predicted RT vs Expt. scan number
Line: Z score of novel peptide
Z: −1.82
count
400
300
200
100
0
−10
−5
0
5
10
Z scores of residuals from best linear fit
